# Analysis and Comparison of Somatic Mutations in Paired Primary and Recurrent Epithelial Ovarian Cancer Samples

**DOI:** 10.1371/journal.pone.0099451

**Published:** 2014-06-17

**Authors:** Yong-Man Kim, Shin-Wha Lee, Sung-Min Chun, Dae-Yeon Kim, Jong-Hyeok Kim, Kyu-Rae Kim, Young-Tak Kim, Joo-Hyun Nam, Paul van Hummelen, Laura E. MacConaill, William C. Hahn, Se Jin Jang

**Affiliations:** 1 Department of Obstetrics & Gynecology, University of Ulsan, ASAN Medical Center, Seoul, Korea; 2 Department of Pathology, University of Ulsan, ASAN Medical Center, Seoul, Korea; 3 Center for Cancer Genome Discovery and Department of Medical Oncology, Dana-Farber Cancer Institute, Boston, Massachusetts, United States of America; 4 ASAN Center for Cancer Genome Discovery, ASAN Medical Center, Seoul, Korea; The Chinese University of Hong Kong, Hong Kong

## Abstract

The *TP53* mutations have been proved to be predominated in ovarian cancer in a study from The Cancer Genome Atlas (TCGA). However, the molecular characteristics of recurrent ovarian cancers following initial treatment have been poorly estimated. This study was to investigate the pattern of somatic point mutations in matched paired samples of primary and recurrent epithelial ovarian cancers, using the OncoMap mutation detection protocol. We have adapted a high-throughput genotyping platform to determine the mutation status of a large panel of known cancer genes. OncoMap v.4.4 was used to evaluate genomic DNA isolated from a set of 92 formalin-fixed, paraffin-embedded (FFPE) tumors, consisting of matched paired samples of initially diagnosed and recurrent tumors from 46 epithelial ovarian cancer (EOC) patients. Mutations were observed in 33.7% of the samples, with 29.3% of these samples having a single mutation and the remaining 4.3% having two or more mutations. Among the 41 genes analyzed, 35 mutations were found in four genes, namely, *CDKN2A* (2.2%), *KRAS* (6.5%), *MLH1* (8.2%) and *TP53* (20.7%). *TP53* was the most frequently mutated gene, but there was no correlation between the presence of mutation in any gene and clinical prognosis. Furthermore, somatic mutations did not differ between primary and recurrent ovarian carcinomas. Every mutation present in recurrent samples was detected in the corresponding primary sample. In conclusion, these OncoMap data of Korean EOC samples provide that somatic mutations were found in *CDKN2A*, *KRAS*, *MLH1*, and *TP53*. No differences in mutational status between primary and recurrent samples were detected. To understand the biology of tumor recurrence in epithelial ovarian cancer, more studies are necessary, including epigenetic modifications or additional mutations in other genes.

## Introduction

Ovarian cancer is the most common cause of death among the gynecologic malignancies [1]. The standard treatment is surgical cytoreduction followed by platinum-based chemotherapy. However, most patients eventually relapse and die of chemo-resistant disease. Unfortunately, available salvage regimens for platinum-refractory ovarian cancer have yielded disappointing results, because response rates are low (20–50%) and the responses are short in duration. It is therefore necessary to examine new treatment strategies for both newly diagnosed patients as well as patients with recurrent cancer [2]. In particular, new means of molecularly and genetically characterizing ovarian cancer are needed in order to personalize and improve treatment [3].

Somatic mutations in oncogenes have been observed in many human cancers and are known to be predictive of drug sensitivity or drug resistance [4]. Somatic mutations more than 30 oncogenes and tumor suppressor genes that have been implicated in ovarian oncogenesis were detected in epithelial ovarian cancer (EOC) samples. Genetic changes drive altered signaling that induces proliferation; inhibits apoptosis; blocks anoikis; increases motility, adhesion, and invasion; and attracts stromal components, including mesenchymal stem cells and blood vessel endothelial cells [5]. This oncogene dependency provides the basis for therapies targeting oncogenes, such as the successful use of imatinib and erlotinib in cancers that harbor *BCL-ABL* and *EGFR* alterations, respectively. However, the mutational spectrum in ovarian cancer is surprisingly simple, as shown in a study from The Cancer Genome Atlas (TCGA). Mutations in *TP53* predominated, occurring in at least 96% of high-grade serous ovarian cancer samples; furthermore, *BRCA1* and *BRCA2* were mutated in 22% of the tumors, owing to a combination of germline and somatic mutations. Seven other significantly mutated genes were identified, but only in 2–6% of high-grade serous ovarian cancer samples [6].

We have adapted a high-throughput genotyping platform to determine the mutation status of a large panel of known cancer oncogenes in order to identify subsets of ovarian cancer patients who might benefit from targeted therapy related to their disease status [7]–[10]. This genotyping platform, called OncoMap, employs mass spectrometric-based genotyping technology (Sequenom) to identify 471 oncogenic mutations in 41 commonly mutated genes (Table S1). This study reports the type and frequency of somatic point mutations in paired primary and recurrent EOC samples using OncoMap.

## Methods

### Patients

The ASAN Center for Cancer Genome Discovery (CCGD), in collaboration with the Dana-Farber Cancer Institute (DFCI), has developed the OncoMap genotyping platform [8]. OncoMap v.4.4 was used to analyze a set of 92 formalin-fixed, paraffin-embedded (FFPE) EOC samples consisting of matched paired samples of initially diagnosed and recurrent tumors from 46 patients treated at the ASAN Medical Center, Korea, from January 2002 to December 2011. All patients had been treated with initial cytoreductive surgery followed by platinum-based chemotherapy and received a second cytoreductive surgery because of recurrent or metastatic disease. All tumor samples were obtained from FFPE tumor specimens based on an 80% cutoff for tumor sample purity. This study was approved by the ASAN Medical Center Institutional Review Board (AMC IRB), and we selected patients who had written the informed consent for using their archival tissues for genetic testing. All data was de-identified.

### DNA Extraction

Review of all H&E slides was performed by a pathologist to confirm the tumor type, and to ensure that the sample was representative of the tumor and that normal surrounding tissues were not included. Genomic DNA was extracted from 10–20 6-µm-thick slides per FFPE block, depending on the tumor size. Purification of genomic DNA was performed using the QIAamp DNA FFPE tissue kit (#56404; Qiagen, Hilden, Germany) according to the manufacturer’s instructions. De-paraffinization with xylene and ethanol was carried out as follows. After incubation for 5 min with 1 ml xylene, the samples were centrifuged for 1 min at 12,000 rpm, and the supernatant was removed without disturbing the pellet. The samples were then washed with 1 ml of absolute ethanol in order to remove the remaining xylene, and the pellet was air-dried for 10 min to allow the residual ethanol to evaporate. The pellet was lysed by incubation with 0.2 mg of proteinase K overnight at 60°C then subjected to column purification. Each genomic DNA sample was eluted in 50 µl of DNase- and RNase-free water, quantified using the Quant-iTTM PicoGreen dsDNA Assay kit (Invitrogen/Life Technologies, Grand Island, NY), and normalized to a 5 ng/µl concentration.

### Genotyping Using OncoMap_V4.4-Core Panel

Profiling of somatic mutations for 41 critical genes related to tumor development was performed using OncoMap version 4.4-Core (OncoMap_v4.4C) under the Sequenom MassARRAY technology platform (Sequenom, San Diego, CA). OncoMap_v4.4C is a multiplex panel of 32 pools of genes that altogether span 471 unique mutation sites in 41 oncogenes and tumor suppressor genes (Table S1) that are known to be druggable targets. This OncoMap panel is an upgraded version of OncoMap_v1, which is described in published literature [7], [8]. A total of 320 ng of purified genomic DNA was used as a template for 32 different pools of multiplex amplification using iPLEX chemistry (#10134–2; Sequenom, San Diego, CA), with 10 ng per reaction. An additional 50–80 ng of DNA was then used for homogenous Mass Extension (hME) validation of mutation candidates that were identified by the iPLEX reaction. Multiplex PCR amplification was performed on 10 ng of genomic DNA in a final volume of 5 µl in a 384-well plate with 0.5 U of HotStarTaq DNA Polymerase (Qiagen), 0.12 µM of each PCR primer, 500 µM of dNTP mix, and 3.5 mM MgCl_2_, using a DNA Engine Dyad Cycler (Bio-Rad, USA). Multiplex PCR was run with the following program: 95°C for 15 min; 45 cycles of 95°C for 20 sec, 56°C for 30 sec, and 72°C for 60 sec; then 72°C for 3 min. Residual deoxynucleotides in the PCR products were inactivated by incubation for 40 min at 37°C with 2 µl of shrimp alkaline phosphatase (SAP) from the iPLEX-Pro kit (#10142-2; Sequenom, San Diego, CA), followed by an additional incubation for 10 min at 85°C to inactivate the SAP. Next, single-base extension (SBE) was performed with an additional 2 ul of iPLEX Gold Chemistry mixture (0.1 µl of iPLEX termination mix, 1.2 µl of extension probe mix, and 0.0205 µl of iPLEX enzyme) as follows: 94°C for 30 sec; 40 cycles of 94°C for 5 sec and five internal cycles of (52°C for 5 sec and 80°C for 5 sec); and 72°C for 3 min. After SBE, 16 µl of DNase-free distilled water was added and desalting was performed by incubation for 25 minutes with 6 mg of cation exchange resin (Sequenom). Finally, 10 nl of the desalted product was spotted onto a 384-format SpectroCHIP II with the MassARRAY Nanodispenser (Sequenom). Mass determination was done with the MassARRAY Analyzer Compact MALDI-TOF mass spectrometer. Genotypes were called using a cluster analysis algorithm developed by the CCGD of DFCI, then reviewed manually by two independent researchers to eliminate any uncertain calls due to clustering artifacts. Sample quality was considered adequate for analysis if more than 80% of the attempted genotypes resulted in identifiable products. For validation of candidate mutations, specific hME genotyping was performed for whole samples. Validation pools for hME were designed using AssayDesigner software in the MassARRAY Typer package (v4.0). Proximal SNPs were filtered, and the specificity of PCR amplification and the subsequent primer extension reaction were confirmed with a maximum of six assays per pool. Multiplex PCR amplification and SAP treatment were conducted in the same manner as for the iPLEX reaction, except that 5 ng DNA was used for the multiplex PCR template. The hME reaction was performed with an additional 2 ul of hME master mix (0.2 µl of appropriate hME EXTEND mix, 1 µl of MassEXTEND primer mix, and 0.025 µl of ThermoSequenase enzyme) as follows: 94°C for 2 min; 75 cycles of 94°C for 5 sec, 52°C for 5 sec, and 72°C for 5 sec; and 72°C for 5 min. The remaining steps, including addition of water, desalting, spotting, and analysis, were performed as for the iPLEX reaction. Only concordant calls from both the iPLEX and the hME analysis were considered to be validated mutations. All detected mutations were confirmed by standard, bidirectional Sanger sequencing.

### Statistical Analysis

The survival analysis was performed using the Kaplan-Meier method and log rank test, and statistical significance was defined as p<0.05. SPSS v21.0 was used for all statistical analyses.

## Results

In this study, 46 pairs of primary-recurrent EOC samples (i.e., 92 EOC samples of which 46 were from primary cancer sites and 46 were from matched paired recurrent sites) were analyzed. All patients had histologically confirmed EOC, of which papillary serous adenocarcinoma was most common (67.4%). All patients except one received adjuvant platinum-based chemotherapy between January 2002 and December 2011. The baseline characteristics of the patient population are summarized in Table 1.

**Table 1:** Clinicopathologic characteristics of patients.

		Total number (N = 46)	Percentage (%)
Age, median (range)		48 (17∼76)	
FIGO stage	I	4	8.7
	II	2	4.3
	III	37	80.4
	IV	3	6.5
Histopathologic type	Serous	31	67.4
	Mucinous	2	4.3
	Endometrioid	4	8.7
	Clear cell	2	4.3
	Transitional cell	1	2.2
	Poorly differentiated	1	2.2
	Mixed†	5	10.9
Residual mass	<1 cm	37	80.4
	≥1 cm	9	19.6
Adjuvant chemotherapy	Paclitaxel + Platinum	34	73.9
	Cyclophosphamide + Platinum	6	13.0
	Docetaxel + Platinum	5	10.9
	Not done	1	2.2
Response to *1°* treatment‡	CR	37	80.4
	PR	4	8.7
	SD	3	6.5
	PD	1	2.2
DFS*, median (range)		22.2 (4.7∼55.4)	
Location of recurrent specimen	Bowel	15	32.6
	Peritoneum	10	21.7
	Liver/Spleen	11	23.9
	Ovary	5	10.9
	Lymph node	2	4.3
	other	3	6.5

† Serous adenocarcinoma and transitional cell carcinoma in 3 cases, serous adenocarcinoma and mucinous carcinoma in 1 case, and transitional cell carcinoma and poorly differentiated carcinoma in 1 case.

‡ CR = complete remission; PR = partial response; SD = stable disease; PD = progressive disease.

*DFS = disease-free-survival.

**Figure 1:**
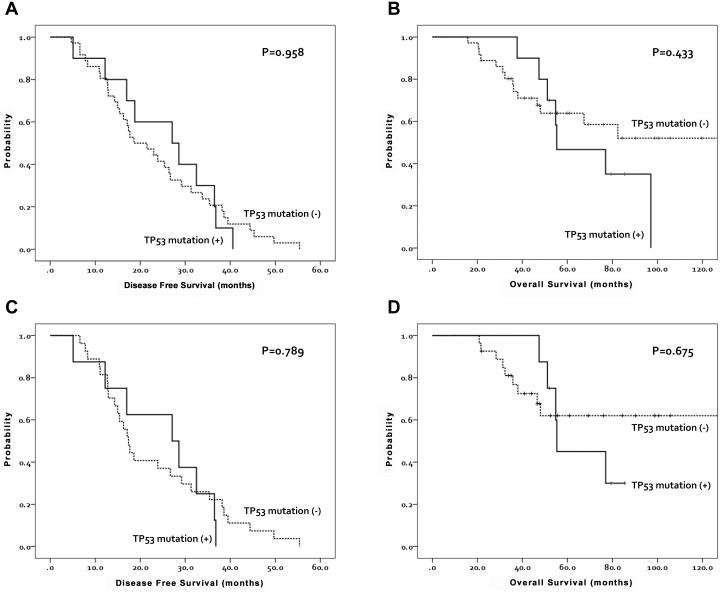
Comparison of disease-free-survival and overall survival with respect to TP53 mutation. Disease-free-survival and overall survival are not different between *TP53* mutation-negative group and *TP53* mutation-positive group. A and B, Disease-free-survival and overall survival in total EOC patients (n = 46, 36 *TP53* mutation (−) vs. 10 *TP53* mutation (+)). C and D, Disease-free-survival and overall survival in serous adenocarcinoma (n = 35, 27 *TP53* mutation (−) serous vs. 8 *TP53* mutation (+) serous).

Of the 92 FFPE samples tested, 35 mutations were identified and validated in 31 samples (33.7%). A single mutation was detected in 29.3% of the samples, and the remaining 4.3% had two or more mutations. The 35 mutations that were validated occurred in four genes, and 19 of these mutations were located in *TP53* (Table 2). Mutations were validated in genes *TP53* (20.7%), *MLH1* (8.7%), *KRAS* (6.5%), and *CDKN2A* (2.2%), with tumor suppressor gene *TP53* the most commonly mutated gene in EOC (20.7% of the samples). This finding is in agreement with two recent reports that demonstrated that *TP53* mutations are the most common somatic gene mutations in high-grade serous ovarian cancer [6], [10]. However, there was no correlation between the presence of a mutation in the *TP53* gene and clinical prognosis in total 46 patients. The median disease-free-survival (DFS) was 20.1 (range, 4.7–55.4) months in the *TP53* mutation-negative group and 27.9 (range, 5.1–40.6) months in the *TP53* mutation-positive group (P = 0.958). The median overall survival (OS) was 47.9 (15.8–177.4) months in the former and 55.0 (37.7–97.1) months in the latter group (P = 0.433) (Fig. 1). The survival analysis between *TP53* wild type and *TP53* mutant type obtained the same result when they only include 31 serous samples (Fig. 1). *TP53* mutations and *MLH1* mutations appeared in papillary serous adenocarcinoma, and otherwise *KRAS* mutations and *CDKN2A* mutations showed up mainly in mucinous adenocarcinoma (Table 3).

**Table 2:** Frequency of mutations.

Gene	Validated mutations	Total (N = 92)	%	Primary (N = 46)	%	Recurrent (N = 46)	%
*CDKN2A*			2.2		2.2		2.2
	CDKN2A_H83Y_c.21961111G>A_h	2	2.2	1	2.2	1	2.2
*KRAS*			6.5		6.5		6.5
	KRAS_G12D_c.25289551C>T_h	4	4.3	2	4.3	2	4.3
	KRAS_G12V_c.25289551C>A_h	2	2.2	1	2.2	1	2.2
*MLH1*			8.7		8.7		8.7
	MLH1_V384D_c.37042244T>A_h	8	8.7	4	8.7	4	8.7
*TP53*			20.7		21.7		19.6
	TP53_R273H_c.7517845C>T_h	2	2.2	1	2.2	1	2.2
	TP53_R273C_c.7517846G>A_h	6	6.5	3	6.5	3	6.5
	TP53_R248Q_c.7518263C>T_h	1	1.1	1	2.2	0	0.0
	TP53_G245S_c.7518273C>T_h	4	4.3	2	4.3	2	4.3
	TP53_R175H_c.7519131C>T_h	6	6.5	3	6.5	3	6.5

**Table 3:** Clinicopathologic characteristics of patients detected somatic mutations.

Case	Age	FIGO Stage	Response to *1°* treatment†	Recurred site	Mutated gene	Sample site (P‡)	Cell type* (P‡)	Sample site (R‡)	Celltype*(R‡)
Case 01	46	IIIc	CR	Colon, LN#, skin, peritoneum	*KRAS*	Ovary	PSA	Skin mass	PSA
Case 02	36	IIIc	SD	Pericardium	*TP53*	Ovary	PSA	Pericardium	PSA
Case 03	44	IIIc	CR	Uterus, colon, small bowel	*MLH1*	Ovary	PSA	Colon	PSA
Case 04	42	IIIc	CR	Liver	*TP53*	Ovary	PSA	Liver	PSA
Case 05	65	IIIc	CR	Paraaortic LN	*TP53*	Omentum	PSA	Paraaortic LN	PSA
Case 06	48	IIIc	CR	Rectum, small bowel	*MLH1*	Ovary	PSA	Rectum	PSA
Case 07	41	IIIc	CR	Peritoneum	*TP53*	Ovary	PSA	Pelvic mass	PSA
Case 08	56	IIIc	CR	Paraaortic LN, pelvic LN	*TP53*	Ovary	PSA	Paraaortic LN	PSA
Case 09	17	IIIb	CR	Ovary, chest wall, peritoneum	*KRAS*	Ovary	MA	Ovary	MA
Case 10	28	Ic	CR	Ovary, diaphragm, omentum	*KRAS, CDKN2A*	Ovary	MA	Ovary	MA
Case 11	56	Ic	CR	Peritoneum	*TP53*	Ovary	EA	Paracolic gutter mass	PSA
Case 12	45	IIc	CR	Small bowel	*TP53*	Ovary	CCC	Small bowel	CCC
Case 13	44	IIIc	CR	Peritoneum, mesentery	*TP53*	Ovary	PSA+TCC	Pelvic mass	PDC
Case 14	60	IIIc	CR	Colon, small bowel	*TP53, MLH1*	Ovary	PSA+TCC	Colon	PSA
Case 15	57	IIIc	CR	Peritoneum	*MLH1*	Ovary	PSA	Hepatic flexure mass	PSA
Case 16	57	IV	CR	Peritoneum	*TP53*	Ovary	PSA	Pelvic mass	PSA

† CR = complete remission; PR = partial response; SD = stable disease; PD = progressive disease.

‡ P means the tumor sample from primary tumor and R means the tumor sample from recurrent tumor.

*PSA = papillary serous adenocarcinoma; MA = mucinous carcinoma; EA = Endometrioid adenocarcinoma; CCC = clear cell carcinoma; TCC = transitional cell carcinoma; PDC = poorly differentiated carcinoma.

# LN = lymph node.

Next, the 46 pairs of primary and recurrent tumor samples were compared in order to evaluate the concordance rate of somatic mutations. Most patients showed the multiple metastases and the tested recurrent tumor samples were obtained from the local recurrence and distant metastasis (Table 3). Interestingly, any somatic mutations occurred in the only recurrent tumor had not detected in this OncoMap analysis of EOC (Table 4). An almost 100% concordance rate was observed when comparing mutations between primary and recurrent tumor pairs. The only one patient (case 12) showed a *TP53* mutation in the primary sample, however, that was not detected in the recurrent sample. Furthermore, the frequency of mutation detection was similar between primary and recurrent tumor. In other words, somatic mutations were not affected by local recurrence or metastasis in EOC.

**Table 4:** Concordance between primary and recurrent paired samples.

Sample†	*CDKN2A*	*KRAS*		*MLH1*	*TP53*				
	CDKN2A_H83Y_c.21961111G>A_h	KRAS_G12D_c.25289551C>T_h	KRAS_G12V_c.25289551C>A_h	MLH1_V384D_c.37042244T>A_h	TP53_R273H_c.7517845C>T_h	TP53_R273C_c.7517846G>A_h	TP53_R248Q_c.7518263C>T_h	TP53_G245S_c.7518273C>T_h	TP53_R175H_c.7519131C>T_h
P1			2.9%						
R1			23.0%						
P2									65.8%
R2									36.7%
P3				78.2%					
R3				70.0%					
P4						62.6%			
R4						31.5%			
P5									54.6%
R5									60.6%
P6				92.2%					
R6				93.9%					
P7					38.8%				
R7					22.5%				
P8								19.0%	
R8								40.6%	
P9		11.5%							
R9		6.4%							
P10	9%	6.0%							
R10	33%	21.9%							
P11						57.2%			
R11						59.9%			
P12							76.0%		
R12							0.0%		
P13								78.9%	
R13								70.3%	
P14				99.5%					19.2%
R14				97.4%					16.0%
P15				57.5%					
R15				46.5%					
P16						60.0%			
R16						62.0%			

† P means the tumor sample from primary tumor and R means the tumor sample from recurrent tumor.

Percentage showed in table 4 means the ratio of mutant allele versus sum of wild and mutation for each position, that is to say the mutant allele frequency.

## Discussion

OncoMap is an optimized mutation profiling platform developed to efficiently analyze mutations in known oncogenes and tumor suppressor genes, many of which are known to predict response or resistance to targeted therapies [8]. Our current version of OncoMap (v.4.4) interrogates 471 mutations in 41 genes that are relevant for cancer. The OncoMap platform can be used to analyze DNA derived from both fresh-frozen tissue and FFPE specimens. The sensitivity and specificity of OncoMap were 93.8% and 100%, respectively, in DNA derived from fresh-frozen tissue, and 89.3% and 99.4%, respectively, in FFPE-derived DNA [8]. When FFPE tissue is used, the sensitivity and specificity of OncoMap are comparable with results using fresh-frozen tissue. OncoMap has not been developed for a specific cancer type, so it has the limitation that contains many genes not to be relevant to a specific biology of each cancer. Nevertheless, the most remarkable advantage of OncoMap is that it enables the screening of hundreds of hotspot mutations from FFPE tissue at a reasonable cost [8]; therefore, this technology could make it easier for clinicians to screen for druggable mutations in diverse cancers. OncoMap has been already been reported as a reliable method for screening for somatic mutations in multiple solid tumor types [7], [9]–[12].

An OncoMap study was performed in EOC to identify mutations that are druggable with novel biologic agents. Although EOC is not characterized by specific gene mutations (except for *TP53*, according to the TCGA data) [6], it is necessary to investigate the somatic mutations associated with differences in disease states. Overall, 31 (33.7%) of 92 FFPE tissues screened using OncoMap had somatic mutations. Of the EOC samples, 20.7% had *TP53* mutations, and fewer samples harbored *MLH1* (8.7%), *KRAS* (6.5%), or *CDKN2A* (2.2%) mutations.

**Figure 2:**
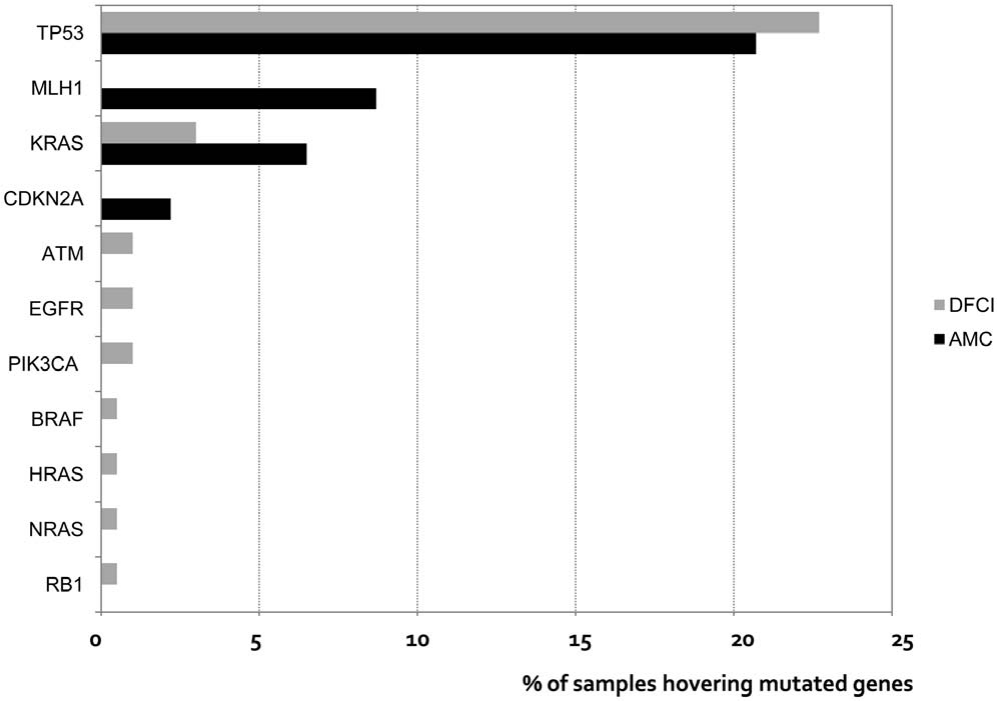
Comparison of somatic mutations between ASAN Medical Center and Dana-Faber Cancer Institute. This OncoMap v.4.4 study from Korean women revealed the similar frequency of *TP53* somatic mutation (20.7% vs. 22.7%) and the distinctive result of *MLH1* mutation (8.7% vs. 0%) comparing previous OncoMap v.3.0 study performed in Dana-Farber Cancer Institute.

*TP53* is a tumor suppressor gene that encodes a protein mediating cell apoptosis, and loss-of-function mutation of *TP53* is one of the most common features of human cancers [13]. From the first comprehensive mapping of the *TP53* mutation rate in a homogeneous group of high-grade serous ovarian cancer patients, the overall *TP53* dysfunction rate approached 100% of 123 patients. Pathogenic *TP53* mutations were identified in 96.7% of these samples by sequencing exons 2–11 and intron-exon boundaries in tumor DNA [14]. Consistent with these published results, *TP53* was mutated in 303 of 316 samples (95.9%) in the TCGA study [6]. The frequency of *TP53* mutation was lower in our study (20.7%) compared to that reported in previous studies. Since OncoMap only investigates *TP53* mutations at seven loci and does not detect deletion events, the lower rate of *TP53* mutations observed in this study is in agreement with recent work from TCGA, as mentioned in another study performed using OncoMap (Fig. 2) [10]. This previous OncoMap study was performed on a set of 203 FFPE advanced staged high grade serous cancer of the ovary specimens in Dana-Farber Cancer Institute, using OncoMap v.3.0. We used an upgraded platform of OncoMap (v.4.4) including more gene and assays and investigated the mutation profile from only Korean women; however, the frequent somatic mutation of EOC was not distinctive comparing previous OncoMap study (Fig. 2). Numerous studies have evaluated the association between *TP53* mutations in ovarian cancer and prognosis. Our data did not show differences in DFS or OS with respect to *TP53* mutation status. Although there have been many reports that the presence of *TP53* mutations is associated with prognosis in ovarian cancer, no association between *TP53* mutation and progression-free or OS was found in the comprehensive mapping of *TP53* mutation in the current study [14]. It is possible that the low number of patients with *TP53* mutations may be insufficient to detect differences in prognosis.

In this study, the second most commonly mutated gene in EOC was *MLH1* (8.7%), which was not found to be mutated in the published OncoMap data in high-grade serous ovarian cancer. Germline mutations in mismatch repair genes, including *MLH1*, *MSH2*, and *MSH6*, have been identified in ovarian cancer patients with hereditary non-polyposis colorectal cancer (HNPCC) [15]. A study that included 1,893 women with EOC suggested that less than 1% of women with ovarian cancer harbor a germline mutation in the HNPCC genes, and pathogenic mutation carriers had an earlier mean age at diagnosis of ovarian cancer, with a greater likelihood of a non-serous histology, and a greater number of relatives with HNPCC-related cancers [15]. In a meta-analysis, somatic mutations in the *MLH1* and *MSH2* genes were found to be prevalent in colorectal cancer, in addition, a higher prevalence of somatic mutations in the *MLH1* gene relative to the *MSH2* gene was observed in the European group [16]. However, there are no reports on *MLH1* somatic mutations in EOC. This is the first report detecting somatic mutations in *MLH1* in sporadic EOC; therefore, there is no independent validation data available. It will be necessary to interrogate whether *MLH1* somatic mutations are associated with specific ovarian cancer types.

*KRAS* mutations (6.5%) and *CDKN2A* mutations (2.2%) were also detected in this study. Mutations in the *KRAS* gene are one of the most frequent genetic abnormalities in ovarian cancer, and are more frequently present in carcinoma of a lower grade and International Federation of Gynecology and Obstetrics (FIGO) stage, and in lesions of a mucinous histotype [17]. *KRAS* mutation is associated with mucinous differentiation, because these mutations also accumulate in mucinous carcinomas of other organs [18], [19]. Three patients had *KRAS* mutations, and that two of them had mucinous malignancies. Furthermore, it has recently been shown that pre-invasive ovarian mucinous tumors are characterized by cyclin-dependent kinase inhibitor 2A (CDKN2A) and Ras pathway aberrations [20]. Notably, the one patient with a *CDKN2A* mutation in our study also had a *KRAS* mutation. This study is the largest and highest-resolution analysis of mucinous benign and borderline tumors carried out to date and provides strong support for the hypothesis that these lesions are precursors of primary ovarian mucinous adenocarcinoma.

We investigated gene mutations in paired samples of initially diagnosed and recurrent tumors from 46 ovarian cancer patients. Many oncologists have been interested in intratumoral heterogeneity as well as the intertumoral heterogeneity between primary and recurrent tumor samples from the same patient. A large prospective study in breast cancer identified a switch in receptor status for ER in 10.2% of patients, for PR in 24.8%, and for HER2 in 2.9%; the switch in receptor status led to a change in the subsequent treatment plan for 17.5% of patients [21]. The authors, therefore, suggested that the management of relapsed breast cancer should include tissue sampling to identify switches in ER, PR, or HER2 status in locally recurrent or metastatic breast cancer, which may influence the planned treatment. Indeed, some reports in lung cancer have demonstrated discordant mutation patterns between primary and metastatic tumors and a heterogeneous distribution of epidermal growth factor receptor (*EGFR*) mutations in individual tumors [22]–[25]. However, heterogeneous mutations in *KRAS* and *TP53* are scarce because these mutations are associated with the early pathogenesis of cancer [26]. In a report examining tumor samples from the Catalogue of Somatic Mutations in Cancer (COSMIC) database and the Aichi Cancer Center Cohort, no discordant mutation patterns were detected among 77 paired primary and metastatic site samples, or among 54 primary and recurrent tumor pairs [27]. The authors concluded that heterogeneous distribution of *EGFR* mutations is rare, but because mutant *EGFR* alleles are selectively amplified within tumors, samples analyzed by less sensitive detection methods may wrongly appear to be heterogeneous for *EGFR* mutations.

In ovarian cancer, some studies have shown discordant expression of genes or protein biomarkers in paired primary and relapsed ovarian cancer tissue samples, as measured by immunohistochemical analyses [28]–[30]. Recently, however, the opposite data has been reported that no new mutations arose from diagnosis to relapse after chemotherapy by exome sequencing of matched samples from one patient, suggesting that mutations already present in the primary tumor contributed to metastasis and chemotherapy resistance [31]. In our study, an almost 100% concordance rate was observed in paired primary and recurrent samples. Although one patient showed discordance, the *TP53* mutation was detected in the primary sample, but not in the recurrent sample. Our findings suggest that these genes (*TP53*, *MLH1*, *KRAS,* and *CDKN2A*) are involved in early tumor development, as mentioned above.

This OncoMap analysis revealed that somatic mutations were rare in EOC. *TP53* mutations were most common, consistent with published OncoMap and TCGA data, and somatic mutations in *CDKN2A*, *KRAS*, and *MLH1* were also detected, although at much lower rates. Since there was no discordance between primary and recurrent samples, we could not find the specific somatic mutation associated to the tumor recurrence and distant metastasis in EOC, among well-known oncogenes and tumor suppressor genes. To understand the biology of tumor recurrence in EOC, more studies including epigenetic modifications or additional mutations in other genes are necessary. Furthermore, future studies will be necessary to correlate the presence of *TP53* mutations with the biologic activity and clinical prognosis of the cancer. In addition, non-serous types of EOC should be analyzed, because molecular events in those cancers may provide an opportunity for treatments targeting specific mutations and pathways. Better functional genetics and disease stratification in ovarian cancer will make novel, targeted therapeutic targets and individualized treatments possible.

## Supporting Information

Table S1 OncoMap_4.4C consists of 439 assays in 32 separate reactions designed to screen 471 mutations in 41critical genes.
(DOC)
