# Supplementary material for: Analysis and Comparison of Somatic Mutations in Paired Primary and Recurrent Epithelial Ovarian Cancer Samples
Source: PLoS One. 2014 Jun 17;9(6):e99451. doi: 10.1371/journal.pone.0099451 (PMC4060993; doi:10.1371/journal.pone.0099451)
Supplement: Table S1 — OncoMap_4.4C consists of 439 assays in 32 separate reactions designed to screen 471 mutations in 41critical genes. (DOC) [file pone.0099451.s001.doc]

Table S1. OncoMap_4.4C gene list

| Gene | Number of mutations |
| --- | --- |
| ABL1 | 16 |
| AKT1 | 1 |
| AKT2 | 2 |
| APC | 13 |
| BRAF | 50 |
| CDK4 | 1 |
| CDKN2A | 11 |
| CSF1R | 7 |
| CTNNB1 | 33 |
| EGFR | 51 |
| ERBB2 | 8 |
| FGFR1 | 2 |
| FGFR2 | 6 |
| FGFR3 | 8 |
| FLT3 | 9 |
| GNA11 | 2 |
| GNAQ | 3 |
| GNAS | 3 |
| HRAS | 16 |
| IDH1 | 3 |
| IDH2 | 2 |
| JAK2 | 1 |
| JAK3 | 3 |
| KIT | 27 |
| KRAS | 24 |
| MAP2K1 | 7 |
| MET | 6 |
| MLH1 | 1 |
| MYC | 6 |
| NPM1 | 3 |
| NRAS | 22 |
| PDGFRA | 20 |
| PIK3CA | 23 |
| PIK3R1 | 14 |
| PTEN | 15 |
| RB1 | 11 |
| RET | 14 |
| SRC | 1 |
| STK11 | 12 |
| TP53 | 7 |
| VHL | 7 |
